# Long-term re-procedure rate after mid-urethral slings for stress urinary incontinence

**DOI:** 10.1007/s00192-019-04223-1

**Published:** 2020-01-20

**Authors:** Sari Tulokas, Päivi Rahkola-Soisalo, Mika Gissler, Tomi S. Mikkola, Maarit J. Mentula

**Affiliations:** 1 Department of Obstetrics and Gynaecology, University of Helsinki and Helsinki University Hospital, PO Box 140, 00029 Helsinki, Finland; 2 National Institute for Health and Welfare (THL), 00300 Helsinki, Finland; 3 Department of Neurobiology, Care Sciences and Society, Karolinska Institute, 17177 Stockholm, Sweden

**Keywords:** Mesh tape, Mid-urethral sling, Stress urinary incontinence, TOT, TVT

## Abstract

**Introduction and hypothesis:**

Long-term safety concerns have risen over the mid-urethral sling operation (MUS) for stress urinary incontinence (SUI), which in some countries has led to litigations and even suspending MUS insertions. We examined the long-term re-procedure rate after MUS operations. The main outcome was re-procedures for SUI. The secondary outcome was surgical interventions due to complications.

**Methods:**

We analysed a retrospective population cohort of 3531 women with MUS operations in 2000–2006 and followed them up until 31 December 2016. Data were collected from a national hospital register and from hospital patient records.

**Results:**

The median follow-up time was 13 years (IQR 11.6–14.8) for the 3280 women with a retropubic MUS (RP-MUS) and 11 years (IQR 10.3–11.9) for the 245 women with a transobturator MUS (TO-MUS). The cumulative number of re-procedures for SUI was 16 (0.5%) at 1 year, 66 (1.9%) at 5 years, 97 (2.8%) at 10 years and 112 (3.2%) at 17 years. This risk was higher after TO-MUS than after RP-MUS operations (OR 3.6, 95% CI 2.5–5.2, *p* < 0.001). The cumulative number of any long-term re-procedure was 43 (1.2%) at year 1, 105 (3.0%) at year 5, 144 (4.1%) at year 10 and 163 (4.6%) at year 17.

**Conclusions:**

Re-procedures occur up to 17 years after primary MUS insertion, but their incidence is low after the first few postoperative years. Re-procedures for recurrent SUI are more common after TO-MUS than RP-MUS.

## Introduction

Urinary incontinence affects up to 40% of women [1], and 10–14% of women are estimated to go through an operation for stress urinary incontinence (SUI) during their lifetime [2, 3]. Studies have proven the mid-urethral sling operation (MUS) to be an effective and safe treatment for SUI in the short- and medium term [4], and it has become the current gold standard treatment for SUI.

However, using surgical mesh in pelvic floor operations has raised concerns about long-term problems such as chronic pain, mesh exposure, dyspareunia, voiding dysfunctions and the need for re-operations to treat complications or recurrent SUI. The US Food and Drug Administration, the Scottish government and the National Health Service (NHS England) concluded in their reviews that complications following these operations are not rare and that more comprehensive evidence on long-term risks of mesh operations is needed [5–7]. In previous cohort studies with follow-up times of up to 10 years, 4.3–4.5% of women received a further surgical treatment for SUI [8, 9], and, including mesh-removal procedures, 4.6–6.9% of women had a re-operation [9, 10].

With our register-based population study, we assess the long-term re-procedure rate after MUS with a follow-up time up to 17 years. The main focus was on re-procedures for SUI, but we also evaluated complications and surgical interventions to treat them. We combined register data with hospital records to determine the complication types and their treatment in detail.

## Methodology

The sample included all retropubic (RP-MUS) and transobturator (TO-MUS) MUS operations performed in the Hospital District of Helsinki and Uusimaa (HUS) from January 1, 2000, to December 31, 2006. This hospital district comprises two university hospitals and five regional hospitals with a population of 1.6 million people as of 2017. Re-operations were included from the index operation until the end of 2016, which resulted in a follow-up time of 10 to 17 years (median 13.2, IQR 11.3–14.7).

We identified the sample women from the HUS hospital records and from the national Care Register for Health Care (Care Register). This register is composed of regulated notifications that every public Finnish health care-providing institute is obligated by law to submit for every in- and hospital out-patient visit. These notifications include admission and discharge dates and all diagnosis and procedure codes. Therefore, the Care Register includes all re-procedures performed in the public sector in Finland. Of all MUS procedures in Finland, 98.4% were performed in the public sector during our study period (National Institute for Health and Welfare). We identified the sample by searching all visits with Nordic Medico-Statistical Committee Classification of Surgical Procedures (NCSP) operation codes for the MUS operation (LEG10, LEG12 and LEG13). The preoperative incontinence type was considered as SUI if the ICD-10 diagnosis code for the operation was N39.3, mixed urinary incontinence (MUI) if it was N39.4 and unknown if any other diagnosis code was used.

**Fig. 1:**
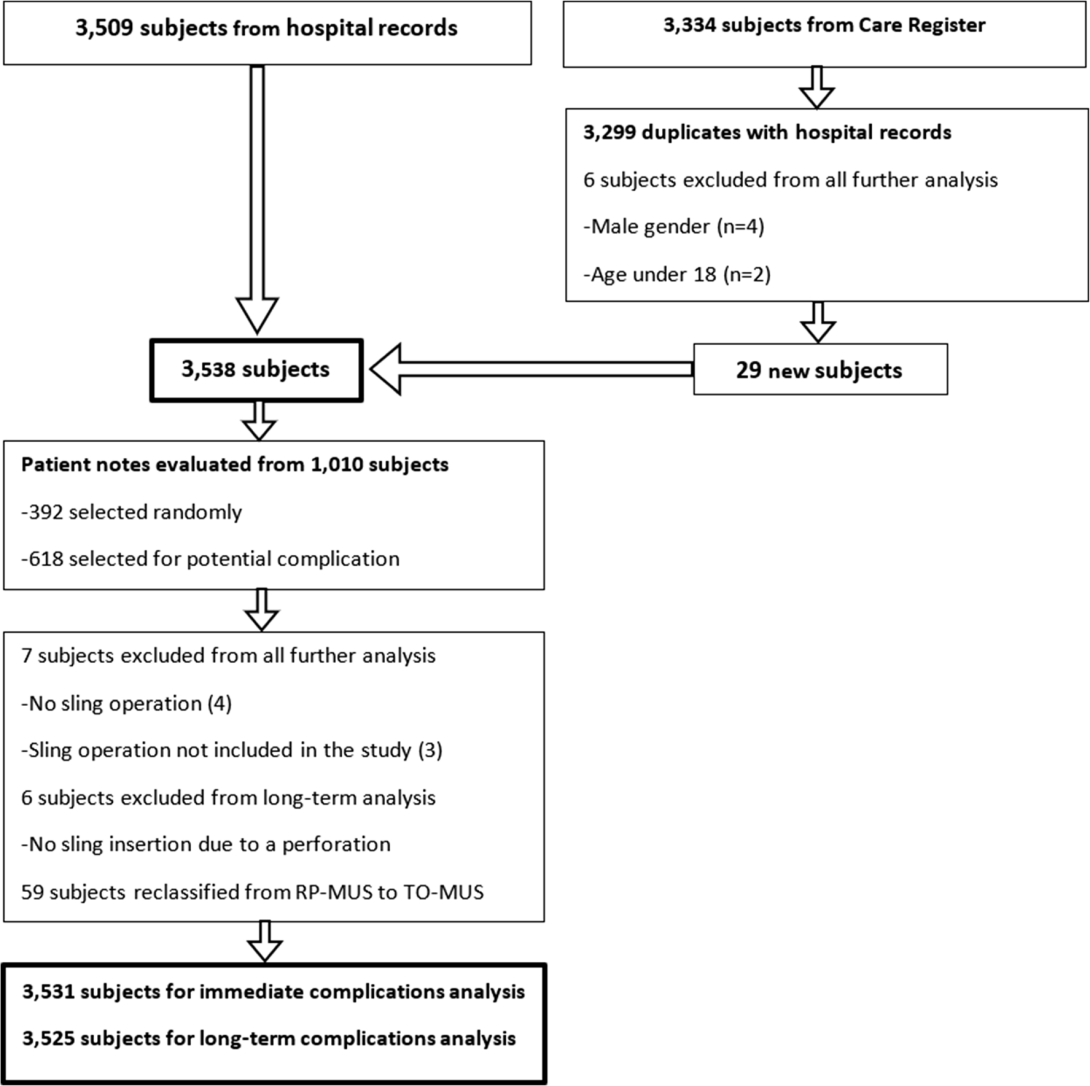
Sample cases obtained from the hospital records and Care Register. *MUS* mid-urethral sling, *Care Register* Care Register for Health Care, *RP-MUS* retropubic mid-urethral sling, *TO-MUS* trans-obturator mid-urethral sling

We identified 3509 women from the hospital records and 3334 from the Care Register.Combined, 3299 (93.1%) of 84 3544 women were found from both sources, 210 (5.9%) only 85 from the hospital records and 35 (1.0%) only from the Care 86 Register; 6 women found only in the Care Register were excluded because of male sex or age < 18 years (Fig. 1). We validated the data on a sample of 1010 women (392 women selected randomly and all the 618 women with a potential complication, see below). We compared their register data and hospital records, which were available for 990 (98.0%) of the selected women. All these women had visits related to urinary incontinence, but we identified four women with a planned MUS operation that was not performed and three women who had undergone a sling operation not included in this study (mini-sling TVT Secur). These seven women were excluded from further analysis. We also identified six cases where the sling was not placed because of an intraoperative bladder perforation. These women were included in the immediate complication analysis but not in the long-term complication analysis. All the remaining 977 women whose patient records were evaluated had a MUS insertion in the index period. After validation, a sample of 3531 women remained for immediate complication analysis and 3525 women for long-term complication analysis. We were able to identify all women who had died during the follow-up time (*n* = 435, 12.3%), but we could not get information on migration to other countries as well as on operations or treatments in another country than Finland.

Complications and re-procedures were identified from the Care Register. The index MUS operation and all subsequent visits were identified, and diagnosis and operation codes were used to define complications (see detailed list of the codes used in Appendix Table 5). Subsequent visits with diagnosis or operation codes clearly indicating that the visit was unrelated to urological or gynecological issues were excluded. To gather more detailed information on the nature and treatment of the complication, we read and evaluated hospital records of the 618 (17.5%) women with a potential MUS complication or re-operation based on the Care Register. For 594 (96.1%) of these women, the patient records were available for the hospital visit and we were able to confirm if they were related to a MUS complication included in this study.

The main outcome, re-procedure for SUI, was defined as a recurrent SUI operation (MUS, urethral bulking injection, colposuspension or bladder neck needle suspension; the two latter were not present among the cohort) ≥ 90 days after the index operation. Only the first re-procedure for SUI was reported for each woman.

Secondary outcomes were complications and re-procedures due to complications. In immediate complications, we included perioperative organ perforations (only bladder and urethra perforation among the cohort), any surgical interventions to treat a complication ≤ 30 days postoperatively and surgical intervention to treat sling exposure 90 days postoperatively. In later complications, we included all sling-related re-procedures that were not already reported in the immediate complications. Exposure was defined as a condition of displaying, revealing or exhibiting mesh or making the mesh accessible [11], and exposures after recurrent MUS operations were also included. The surgical treatment of MUS exposure was divided into sling reburial (covering the exposed sling without resecting the sling), partial sling resection (exposed sling excised without intercepting the sling) and sling resection (exposed sling excised intercepting the sling). Sling cut was defined as an incision of the sling into two pieces in the absence of sling exposure and sling mobilizing as loosening the sling without resecting it. Tissue exploration or laparoscopy to remove sling material as completely as possible was referred to as sling removal.

The National Institute for Health and Welfare of Finland authorized the use of the Care Register data (THL/958/5.05.00/2017), and HUS authorized the use of hospital records (HUS/138/2017). The register authorities assessed the ethics of the study and, as no contact with the subjects was included, the study was exempted from evaluation by an Ethics Committee. In advance, we performed a preliminary estimation of the sample size.

IBM SPSS Statistics 25 was used for statistical analysis. To compare groups, we used the Student’s t-test for continuous variables and the chi-squared or Fisher’s exact test, when appropriable, for categorical variables. To calculate confidence intervals, we used Clopper-Pearson for binomial variables and Student’s t-test for continuous variables. We used Kaplan-Meier to estimate survival and Cox regression to analyse hazard ratios (HR). We used odds ratio (OR) to assess the association between re-operations and index MUS type, Kaplan-Meier to estimate survival and Cox regression to analyse hazard ratios (HR).

## Results

The study included 3531 women: 3286 (93.1%) with RP-MUS and 245 (6.9%) with TO-MUS operations (Table 1). The median follow-up time was 13.2 years (IQR 11.3–14.7) for the whole sample: 13 years (IQR 11.6–14.8) for the 3280 women with RP-MUS and 11 years (IQR 10.3–11.9) for 245 women with TO-MUS. The median age (58 years; IQR 50–67) did not differ between the groups. Most of the operations were performed for SUI in both groups, while concomitant operations were more common in women with RP-MUS (7.7% vs. 3.7%, *p* = 0.02).

**Table 1:** Demographics of the 3531 women undergoing a MUS operation during 2000 to 2006

	All MUS (3531)	RP-MUS (3286)	TO-MUS (245)	*p* value
Age, mean in years (±SD)	58.5 (11.6)	58.5 (11.6)	58.5 (12.0)	1.0
Urinary incontinence type, number (%)				< 0.001
Stress urinary incontinence	2961 (83.9)	2867 (87.2)	94 (38.4)	
Mixed urinary incontinence	367 (10.4)	345 (10.5)	22 (9.0)	
Undefined	203 (5.7)	74 (2.3)	129 (52.7)	
Concomitant operations, number (%)				
Any operation	264 (7.5)	255 (7.8)	9 (3.7)	0.02
Any pelvic organ prolapse operation	169 (4.8)	164 (5.0)	5 (2.0)	0.04
Anterior colporraphy	48 (1.4)	47 (1.4)	1 (0.4)	0.3
Posterior colporraphy	74 (2.1)	72 (2.2)	2 (0.8)	0.2
Hysterectomy	25 (0.7)	24 (0.7)	1 (0.4)	1.0
Operating hospital, number (%)				< 0.01
University clinics	1898 (53.8)	1729 (52.6)	169 (69.0)	
Regional clinics	1633 (46.2)	1557 (47.4)	76 (31.0)	
Operation year, number (%)				0.02
2000	412 (11.7)	412 (12.5)	–	
2001	445 (12.6)	445 (13.5)	–	
2002	669 (18.9)	669 (20.4)	–	
2003	564 (16.0)	559 (17.0)	5 (2.0)	
2004	511 (14.5)	454 (13.8)	57 (23.3)	
2005	503 (14.2)	434 (13.2)	69 (28.2)	
2006	427 (12.1)	313 (9.5)	114 (46.5)	
Median follow-up time, years (IQR)	13.2 (11.3–14.7)	13.4 (11.6–14.8)	11.0 (10.3–11.9)	
Alive at follow-up end (%)	3090 (87.7)	2869 (87.5)	221 (90.2)	0.21

*MUS* mid-urethral sling, *RP-MUS* retropubic mid-urethral sling, *TO-MUS* trans-obturator mid-urethral sling

Immediate complications occurred in 76 (2.2%) MUS operations (Table 2). This rate did not differ (*p* = 0.4) between the two groups (2.1% for RP-MUS and 2.4% for TO-MUS). Bladder perforation was the most common complication (*n* = 41; 1.2%) and it occurred only in the RP-MUS group, as did all heavy bleedings that led to a laparotomy (*n* = 6, 0.2%). Immediate sling exposures took place more often with TO-MUS (2.0% vs. 0.4%; OR = 4.6, 95% CI 2.2–9.6 with *p* = 0.05). The detailed data on immediate complications are found in Table 2.

**Table 2:** Immediate complications after the MUS operations (*n*, %, 95% CI) and odds ratios (95% CI)

	All MUS (*n* = 3531)	RP-MUS (*n* = 3286)	TO-MUS (*n* = 245)
Any complication	76 (2.2, 1.7–2.7)	70 (2.1, 1.7–2.7)	6 (2.4, 0.9–5.3)
Odds ratio	–	–	1.1 (0.5–2.5)
Any immediate re-operation	36 (1.0, 0.7–1.4)	30 (0.9, 0.6–1.3)	6 (2.4, 0.9–5.3)
Odds ratio	–	–	2.4 (1.2–5.1)
Perforations, *n* (%, 95% CI)	42 (1.2, 0.9–1.6)	42 (1.3, 0.9–1.7)	–
Bladder	41	41	–
Urethra	1	1	–
No MUS due to perforation	6	6	–
Perforation detected postoperatively	4	4	–
Any sling-specific complication	26 (0.7, 0.5–1.1)	20 (0.6, 0.4–0.9)	6 (2.4, 0.9–5.3)
Exposure	16 (0.5, 0.3–0.7)	11 (0.3, 0.2–0.6)	5 (2.0, 0.7–4.7)
Odds ratio	–	–	4.6 (2.2–9.6)
Exposure treatment			
-Sling reburied	3	1	2
-Sling partially resected	2	2	–
-Sling resected	6	4	2
-More than one surgical intervention	2	1	1
-Treatment unknown	3	3	–
Sling cut or mobilized	6 (0.2)	5 (0.2)	1 (0.4)
Voiding difficulties	2	2	–
Pain	1	1	–
Reason unknown	3	2	1
Sling removed	5 (0.1)	5 (0.2)	–
Perforation detected postoperatively	4	4	–
Fistula	1	1	–
Laparotomy due to heavy bleeding	6 (0.2)	6 (0.2)	–
Additional suture due to bleeding	1 (0.03)	1 (0.03)	–
Draining of haematoma or abscess	3 (0.1)	3 (0.1)	–

*MUS* mid-urethral sling, *RP-MUS* retropubic mid-urethral sling, *TO-MUS* trans-obturator mid-urethral sling

**Fig. 2:**
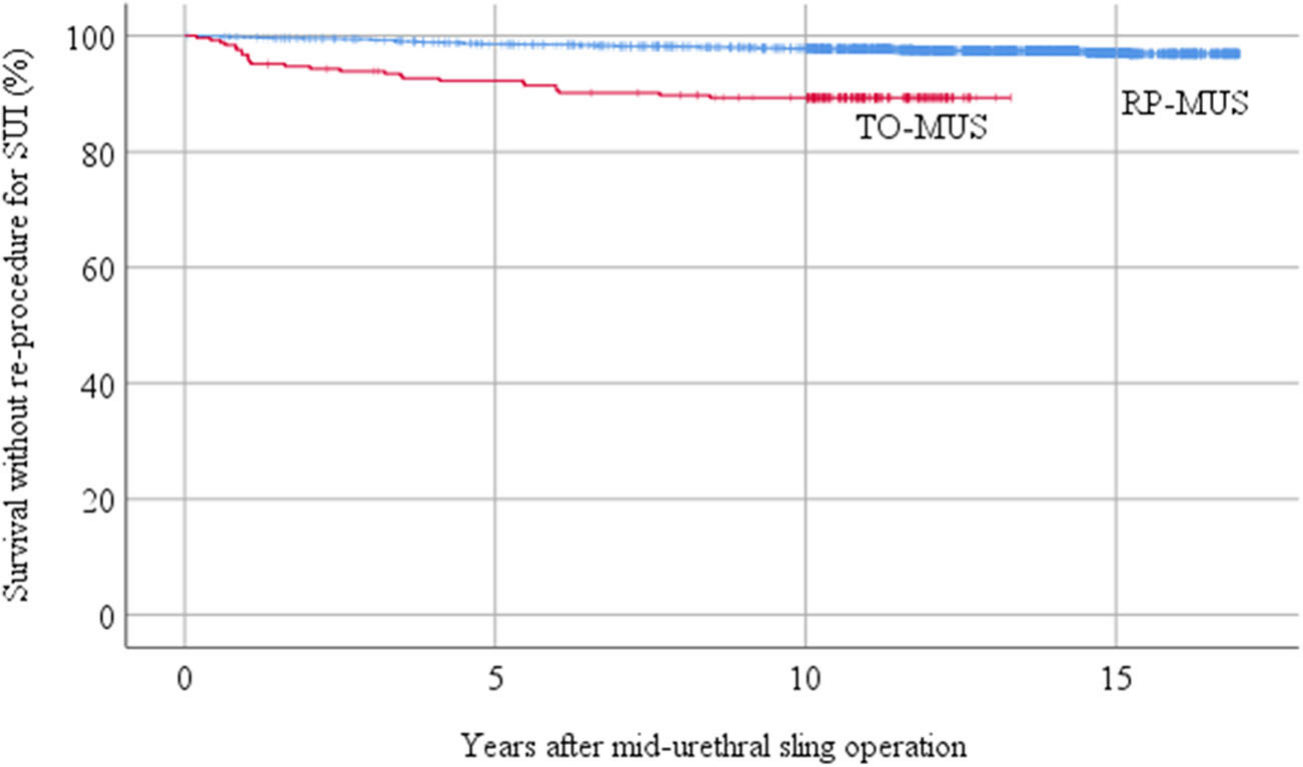
Survival without re-procedure for SUI (%)

Re-procedures for SUI were performed on 112 women (3.2%): 52 (46.4%) new RP-MUS operations, 20 (17.9%) new TO-MUS operations, 38 (33.9%) urethral bulking injections with Bulkamid® and 2 (1.8%) urethral bulking injections with Zuidex® (Table 3). The cumulative number of new SUI procedures was 16 (0.5%) at 1 year, 66 (1.9%) at 5 years, 97 (2.8%) at 10 years and 112 (3.2%) at 17 years (Fig. 2). The risk for a new SUI procedure was higher in the TO-MUS group than in the RP-MUS group (OR 3.6, 95% CI 2.5–5.2 with *p* = 0.05), and the median time until re-procedure was shorter in the TO-MUS group (1.8 years) than in the RP-MUS group (4.6 years, *p* = 0.008) (Table 3). This difference persisted even if the risk was adjusted for the year of operation, incontinence type, immediate exposure and age (Table 4). Women with mixed urinary incontinence and immediate complications had a significantly greater risk for a new SUI procedure.

**Table 3:** Long-term complications after MUS operations (*n*, %, 95% CI) and odds ratios (95% CI)

	All MUS (3525)	RP-MUS (3280)	TO-MUS (245)
Any re-procedure	163 (4.6, 0.4–5.4)	134 (4.1, 3.4–4.8)	29 (11.8, 8.1–16.6)
Odds ratio	–	–	2.8 (1.9–3.9)
Years to re-procedure, median in years (IQR)	3.0 (0.8–6.9)	3.5 (1.0–7.9)	1.6 (0.7–4.8)
Re-procedure for SUI	112 (3.2, 2.6–3.8)	86 (2.6, 2.1–3.2)	26 (10.6, 7.1–15.2)
Odds ratio	–	–	3.6 (2.5–5.2)
Years to re-procedure, median (IQR)	4.1 (1.7–7.7)	4.6 (3.0–8.8)	1.8 (0.9–5.5)
Re-procedure type			
New RP-MUS	52 (46.4)	33 (38.4)	19 (73.1)
New TO-MUS	20 (17.9)	20 (23.3)	0
Urethral bulking injection	40 (35.7)	33 (38.4)	7 (26.9)
Sling cut, resected or removed before re-procedure for SUI	21 (18.8)	17 (19.8)	4 (15.4)
Previous incontinence operation before index MUS operation	12 (13.5)	10 (15.2)	2 (8.7)
Surgical intervention other than re-procedure for SUI	75 (2.1, 1.7–2.7)	70 (2.1, 1.7–2.7)	5 (2.0, 0.7–4.7)
Odds ratio	–	–	1.0 (0.4–2.3)
Years to surgical intervention, median (IQR)	1.4 (0.5–5.9)	1.6 (0.5–6.4)	0.6 (0.3–2.5)
Previous incontinence operation before index MUS operation	4 (7.5)	2 (4.1)	2 (50.0)
Any sling-specific surgical intervention	71 (2.0, 1.6–2.5)	66 (2.0, 1.6–2.6)	5 (2.0, 0.7–4.7)
Odds ratio	–	–	1.0 (0.4–2.4)
Years to surgical intervention, median (IQR)	1.4 (0.5–5.9)	1.6 (0.5–6.4)	0.6 (0.3–2.5)
Exposure	46 (1.3, 1.0–1.7)	41 (1.3, 0.9–1.7)	5 (2.0, 0.7–4.7)
New MUS before exposure	16 (27.1)	14 (26.4)	2 (33.3)
Exposure location			
-Vagina	42 (91.3)	38 (92.7)	4 (80.0)
-Bladder	1 (2.2)	1 (2.4)	0
-Urethra	1 (2.2)	1 (2.4)	0
-Unknown	2 (4.3)	1 (2.4)	1 (20.0)
Exposure treatment			
Sling re-buried	2 (4.3)	1 (2.4)	1 (20.0)
Sling partially resected	9 (19.6)	9 (22.0)	0
Sling resected	27 (58.7)	23 (56.1)	4 (80.0)
Multiple surgical interventions for exposure	8 (17.4)	8 (19.5)	0
Sling removal	6 (0.1, 0.0–0.3)	6 (0.2, 0.1–0.4)	0
Reason for sling removal			
Chronic infection and fistula	3 (50.0)	3 (50.0)	0
Chronic infection without fistula	1 (16.7)	1 (16.7)	0
Exposure in bladder or urethra	2 (33.3)	2 (33.3)	0
Sling cut	25 (0.7, 0.5–0.1)	25 (0.8, 0.5–1.1)	0
Reason for sling cutting			
Voiding difficulties	14 (56.0)	14 (56.0)	0
Pain	3 (12.0)	3 (12.0)	0
Urge symptoms	3 (12.0)	3 (12.0)	0
Unknown	5 (20.0)	5 (20.0)	0
Granuloma removed, *n* (%)	4 (0.1)	4 (0.1)	0
Abscess drained, *n* (%)	3 (0.1)	3 (0.1)	0

*MUS* mid-urethral sling, *RP-MUS* retropubic mid-urethral sling, *TO-MUS* trans-obturator mid-urethral sling, *SUI* stress urinary incontinence

**Table 4:** Risk factors for re-procedure for SUI

Univariate analysis	HR	95% CI, *p* = 0.05		*p* value
Age (continuous)	1.0	1.0	1.0	0.10
Operation type				
RP-MUS	1 (ref)			
TO-MUS	4.8	3.1	7.5	< 0.01
Year				
2000 to 2003	1 (ref)			
2004 to 2006	2.3	1.6	3.4	< 0.01
Incontinence type				
Stress urinary incontinence	1 (ref)			
Mixed and other urinary incontinence	1.8	1.1	3.0	0.02
Immediate complications				
Any immediate complication	6.5	3.5	12.1	< 0.01
Immediate exposure	23.9	11.6	49.2	< 0.01
Perforation	1.8	0.4	7.3	0.4
TO-MUS adj. with operation year				< 0.01
2004 to 2006 (ref: 2000 to 2003)	1.7	1.1	2.6	0.02
Multivariate analysis	HR	95% CI, *p* = 0.05		*p* value
TO-MUS adj. with operation year, incontinence type, immediate exposure and age				< 0.01
2004 to 2006 (ref: 2000 to 2003)	1.6	1.1	2.5	0.03
Mixed and other urinary incontinence (ref: SUI)	1.8	1.1	3.0	0.02
Immediate exposure	13.7	6.2	30.0	< 0.01
Age (continuous)	1.0	1.0	1.0	0.14
TO-MUS (ref: RP-MUS)	3.2	1.9	5.4	< 0.01

*SUI* stress urinary incontinence, *RP-MUS* retropubic mid-urethral sling, *TO-MUS* trans-obturator mid-urethral sling

In long-term re-operations for MUS complications (*n* = 75; 2.1%), exposure was the most common cause: 46 cases in the RP-MUS group (1.4%) and 5 cases in the TO-MUS group (2.0%). The sling was cut in 25 (0.7%) RP-MUS: 14 (56%) for voiding difficulties, 3 (12%) for pain, 3 (12%) for urge symptoms and 5 (20%) for unknown reasons, while no TO-MUS slings were cut in the long term (Table 3). The sling was removed in six (0.2%) women due to either an infection or sling exposure in the bladder or urethra.

The cumulative number of any long-term re-operation after MUS was 43 (1.2%) at year 1, 105 (3.0%) at year 5, 144 (4.1%) at year 10 and 163 (4.6%) at year 17. At 13 years, the end of TO-MUS follow-up time, the rate for any long-term reoperation was 3.5% for RP-MUS operations and 11.8% for TO-MUS operations. At 17 years, the end of the RP-MUS follow-up time, the rate for any long-term reoperation was 4.1% for the RP-MUS women. The overall rate for immediate complications or any long-term re-operation was 5.2% (*n* = 184) at the end of the follow-up time.

## Discussion

In a cohort of 3525 women with a follow-up time up to 17 years after a MUS operation, there were few (3.2%) re-operations due to recurrent SUI. Even though re-operations continued to occur throughout the follow-up period, the incidence was moderate after the first few postoperative years. At the end of the follow-up, 4.6% of the women had gone through a long-term re-operation, most of which were re-operations for SUI.

Almost all immediate complications, except sling exposure, followed only RP-MUS operations. Even though bladder perforation was the most common immediate complication, the occurrence was still low (1.2%) compared with the bladder perforation rate of 4.5% reported in the Cochrane review and the rate of 3.8% in a Finnish national cohort study [4, 12]. However, bladder perforation does not usually require a new operation when detected intraoperatively, and it did not increase the re-operation risk, whereas any immediate complication and immediate sling exposure did.

Our results concur with previous long-term studies that have reported a higher risk for SUI re-operation after TO-MUS compared with RP-MUS [8, 13]. The steep V-shaped angle of the tape in the RP-MUS operation may enhance the long-term efficacy, and the latest NHS guideline recommends using RP-MUS in standard situations [14]. However, the re-operation rates after RP-MUS and TO-MUS differed already in the first few postoperative years, unlike in previous short- and mid-term studies [4], and their difference was also larger than in previous long-term studies [8, 13]. Preceding sling cut or removal or previous incontinence operations cannot explain this difference in re-operation risk because they were more common with RP-MUS. Of the 31 slings that were cut in our study, only one occurred in the TO-MUS group; the most common indication was voiding difficulties, which is known to be more common after RP-MUS than TO-MUS [4]. A learning curve is a plausible partial reason for the deviance in the re-procedure rate; TO-MUS was first introduced in Finland during our study period without strict operator restrictions, whereas performing RP-MUS independently required an obligatory training period. However, the learning curve cannot fully explain the difference because the higher risk for re-operation for SUI persisted when only the year 2006 was considered (OR 3.3, 95% CI 1.8–6.1). Nevertheless, our total rate (3.2%) for SUI re-operations was comparable to that of the previous retrospective long-term cohort studies (1.9 to 4.5%) [8, 10, 15]. With these retrospective data, we cannot determine how the type (RP-MUS versus TO-MUS) of the index operation or re-procedure for SUI was selected.

In other long-term re-operations, the most common was a sling-specific surgical intervention (2.9%). This figure is in line with previous studies that reported a 3.3% rate for partial or total sling removal at 9 years [8] and 2.8% rate for sling shortening, reburying, incision or excision [15]. In our study, exposure was the most common reason for sling resections and for all sling revisions. Sling reburial and resection without cutting the sling carried a risk for a new sling revision; for 15.7% with exposure, a second procedure was needed after the initial sling reburial or resection. However, as sling cutting and removal is associated with up to 61% risk for SUI symptom relapse, minimal mesh revision may be reasonable [16]. Similar to re-operations for SUI, any other surgical intervention took place earlier and was more common with TO-MUS women than RP-MUS women. This is in line with the previously reported lower sling removal rate after RP-MUS vs. TO-MUS (HR 0.72, 95% CI 0.62–0.84) [8].

The strength of our study was that we were able to check the data validity in our sample: 99.3% of the women had a MUS operation at the target period, and the re-operations checked from patient records had been reported accurately. Our sample and outcomes are clinically representative because of the unselected population that includes 25% of all MUS operations performed in Finland in 2006 (National Institute for Health and Welfare). A further strength of our study is the long follow-up time of up to 17 years. Long follow-up time is needed to assess the lifetime risks for re-operation after MUS operation for recurrent SUI or MUS-related complication, which continued to occur throughout our follow-up time, although at a moderate rate.

The main limitation of our study was the inability to assess the incidence of recurrent SUI, pain, dyspareunia and lower urinary tract symptoms problems unless they were treated surgically. However, surgical intervention is a very robust and thus reliable end point to detect a recurrent SUI, as well as other complications. In addition, we were not able to completely determine some important patient characteristics, such as previous incontinence operations, BMI, comorbidities and smoking status. However, when we compared the patient records of women with re-operations with a group of 139 randomly selected patients, there were no significant differences in the rate of previous incontinence operations.

In conclusion, our results suggest that RP-MUS has a better long-term efficacy than TO-MUS in treating SUI. In our data, RP-MUS included a higher risk for bladder perforation and re-procedure for heavy bleeding and voiding difficulties, and TO-MUS included a higher re-procedure risk for mesh exposure. Long-term re-operations for MUS complications can occur, but the incidence is low after the first few postoperative years.

The use of MUS procedure is now under re-evaluation in many countries. In our view, the results of our study show an acceptable risk level for long-term complications. For clinicians who perform this operation, these results help to inform their patients of long-term re-operation risk.

## Appendix

**Table 5:** Diagnosis codes (ICD-10) and operation codes (NCSP) found in the cohort and used to identify visits for MUS complication

ICD-10	NOMESCO
N30* Cystitis N31* Neuromuscular dysfunction of bladder, not elsewhere classified N32* Other disorders of bladder N33* Bladder disorders in diseases classified elsewhere N34* Urethritis and urethral syndrome N35* Urethral stricture N36* Other disorders of urethra N37* Urethral disorders in diseases classified elsewhere N39.0 Urinary tract infection, site not specified N39.3 Stress incontinence N39.4 Other specified urinary incontinence N99* Intraoperative and postprocedural complications and disorders of genitourinary system, not elsewhere classified N99.6 Intraoperative haemorrhage and haematoma of a genitourinary system organ or structure complicating a procedure R30* Pain associated with micturition R31* Haematuria R32* Unspecified urinary incontinence R34* Anuria and oliguria R36* Urethral discharge R39* Other and unspecified symptoms and signs involving the genitourinary system R52* Pain, unspecified T81* Complications of procedures, not elsewhere classified T83* Complications of genitourinary prosthetic devices, implants and grafts	KWE* Reoperation for deep haemorrhage in urological surgery LEG96 Other vaginal operation for incontinence LEG00 Vaginal urethrocystorrhaphy LEG20 Plastic repair of female pelvic floor with levator division KDG* Operations on female urethra and bladder neck for urinary incontinence KDV20 Submucous urethral injection KDV22 Transluminal endoscopic submucous urethral injection LWL03 Reoperation for gynaecological pelvic organ prolapse mesh KDH* Reconstructive operations on urethra KDW* Other operations on urethra KKF* Removal of foreign body from retroperitoneal space KCF* Removal of foreign body from bladder KKW* Other operations on retroperitoneal space KCH* Reconstructive operations on bladder KCW* Other operations on bladder KWW* Other reoperation in urological surgery
